# Adherence to Dietary Behavior Recommendations Moderates the Effect Between Time Since Metabolic and Bariatric Surgery and Percentage Total Weight Loss

**DOI:** 10.1007/s11695-024-07359-2

**Published:** 2024-06-18

**Authors:** Alexander Bäuerle, Matthias Marsall, Marco Niedergethmann, Maximilian Freiherr von Feilitzsch, Anna-Lena Frewer, Eva-Maria Skoda, Sjaak Pouwels, Till Hasenberg, Martin Teufel

**Affiliations:** 1 Clinic for Psychosomatic Medicine and Psychotherapy, University of Duisburg-Essen, LVR-University Hospital Essen, Virchowstr. 174, 45147 Essen, Germany; 2 Center for Translational Neuro- and Behavioral Sciences (C-TNBS), University of Duisburg-Essen, Virchowstr. 174, 45147 Essen, Germany; 3 Institute for Patient Safety (IfPS), University Hospital Bonn, 53127 Bonn, Germany; 4 Department of Surgery, Obesity and Metabolic Surgery Center, Alfried Krupp Hospital Essen, 45131 Essen, Germany; 5 Clinic for General, Visceral and Minimally Invasive Surgery, Heinrich Braun Clinic Non-Profit GmbH, Academic Teaching Hospital of the University of Leipzig and the University Hospital Jena, 08060 Zwickau, Germany; 6 Department of Intensive Care Medicine, Elisabeth-Tweesteden Hospital, Tilburg, The Netherlands; 7 Helios Obesity Center West, Helios St. Elisabeth Hospital Oberhausen, Witten/Herdecke University, Helios University Hospital Wuppertal, 42283 Wuppertal, Germany

**Keywords:** %TWL, Rebound, Dietary Behavior, Adherence, MBS, Lifestyle, Moderation Analysis, Interaction Effect

## Abstract

**Purpose:**

Metabolic and bariatric surgery (MBS) is the gold standard in treating severe obesity. Previous research implies that different psychological and behavior-related factors might be critical for MBS’ sustained success. Yet adherence to dietary behavior recommendations and its impact on weight development is rarely examined. This study investigated the relationship between adherence to dietary behavior recommendations and the percentage of total weight loss (%TWL) after MBS.

**Materials and Methods:**

This study is a cohort study (acquisition in Germany). *N* = 485 patients after MBS, being in grade III of obesity (body mass index (BMI) ≥ 40 kg/m^2^) pre-MBS, were included. Participants answered a standardized assessment on the relevant constructs, including adherence to dietary behavior recommendations, depression symptoms, weight, diet, and MBS characteristics.

**Results:**

BMI pre-MBS, type of MBS, age, regularity of physical activity, and depression symptoms were identified as significant covariates of %TWL and adherence. Within 6 months after MBS, adherence seems to peak, *F*_(5,352)_ = 12.35, *p* < .001. Adherence and time since MBS predict %TWL. A higher adherence (moderator) is related to a higher %TWL, *R*^2^ = 52.65%, *F*_(13,344)_ = 31.54, *p* < .001.

**Conclusion:**

After MBS, adherence to dietary behavior recommendations seems crucial for maximizing its success. Implications for the optimization of MBS’ success in aftercare management arise. In particular, behavior modification interventions should be routinely implemented.

**Graphical Abstract:**

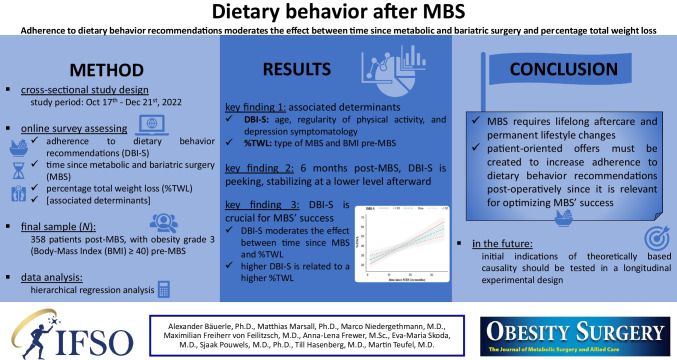

**Supplementary Information:**

The online version contains supplementary material available at 10.1007/s11695-024-07359-2.

## Introduction

Obesity is defined as abnormal or excessive body fat, where excess energy is stored as fat [1]. Despite multifactorial causes, obesity usually results from an imbalanced energy budget: calorie intake > expenditure [1]. Comorbid physical diseases (e.g., type 2 diabetes, ischemic and hypertensive heart disease) and mental diseases (e.g., depression, binge eating disorder) often occur, leading to a significant reduction in quality of life [2]. Especially, people with severe obesity (BMI ≥ 40 kg/m^2^) have a higher mortality (12-fold) [3].

Metabolic and bariatric surgery (MBS) is considered the gold standard for treating patients with obesity (BMI ≥ 35 kg/m^2^) and severe obesity (BMI ≥ 40 kg/m^2^) [4, 5]. In 2021, the most common surgical therapy options worldwide were sleeve gastrectomy (55.4%), Roux-en-Y gastric bypass (29.3%), and omega loop bypass (6.6%) [6]. However, MBS’ success has some shortcomings. A large proportion of patients experience weight regain [7]. Weight regain is generally defined as a renewed weight gain after an initially successful weight loss [8], usually occurring 18–24 months after MBS [7]. It can pose the same health risks as severe obesity and indicates a need for treatment [9]. Obesity’s comorbid diseases can lead to the unsustainable success of MBS [2].

The WHO [3] identified “sedentary lifestyles” (e.g., physical inactivity, smoking) in industrialized countries as the main reason for people becoming obese, while previous research suggests that adverse outcomes of MBS (e.g., weight regain) can also be attributed to lifestyle [10]. There are several determinants (e.g., low socioeconomic status, psychosocial circumstances, dietary and physical activity behaviors, quality of care transition, patient empowerment, compliance/adherence) for one’s weight development post-MBS [2, 10, 11]. Ultimately, a permanent (dietary and behavioral) lifestyle change is most important for MBS’ success [12, 13]. Empirically, some findings highlight that patients’ dietary behavior, encompassing the categories “food choice, eating behavior, and dietary intake/nutrition” [14], is especially critical for MBS’ success [15, 16]. In detail, adverse outcomes can be attributed to sedentary lifestyles and non-adherence to strict dietary recommendations [9]. This is contrasted with the fact that to prevent weight (re)gain, one must reduce fat intake [3]. The Dietary Behavior Inventory-Surgery (DBI-S) is a validated instrument assessing adherence to dietary behavior recommendations post-MBS [17].

### Purpose/Aims

This study aimed to understand the relationship between adherence to dietary behavior recommendations and %TWL after MBS.

Firstly, this study investigated how adherence to dietary behavior recommendations changes after MBS. Secondly, the role of adherence to dietary behavior recommendations in patients’ %TWL after MBS was examined.

## Materials and Methods

### Study Design

Via Unipark (Tivian XI GmbH) [18], an online survey software, data were collected between October 17 and December 21, 2022, in a cross-sectional study. Participants were acquired at the Department of Surgery, Obesity and Metabolic Surgery Center in Essen, Germany, and the Helios Obesity Center West in Oberhausen, Germany, as well as via an online flyer in topic-related social media groups.

The Ethics Committee of the Medical Faculty of the University of Duisburg-Essen approved the conductance of the study (file number 20–9718-BO).

### Participants

Participants had to be of legal age (18 + years), German-speaking, have undergone MBS in the past 3 years, and have agreed to an electronic informed consent form. Participants remained anonymous and received no monetary incentive. Their participation was voluntary and terminable at any time. Weight regain usually occurs 18–24 months, sometimes even more than 2 years, after MBS [7]. Accordingly, a period of more than 2 years after MBS seems adequate for validly assessing weight regain. Considering even longer periods of time might lead to heterogeneous outcomes.

To further improve data quality, 485 participants were reduced to a total sample of *N* = 358 after the exclusion of individuals with BMI < 40 before MBS (*n* = 30) and < 18.5 after MBS (*n* = 1), below average questionnaire completion time (*M* − 1 SD,* n* = 73), and incomplete DBI-S score (*n* = 23). A comparison of the sociodemographic data of the final sample and excluded participants showed one significant difference regarding participants’ occupational status (see Table S1). The average survey completion time was 20.50 min (SD = 10.79).

### Assessment Instruments

All assessment instruments were based on self-reports. In addition to sociodemographic characteristics (age, gender, height, marital status, educational degree, occupational status), weight-/MBS-related (BMI grade before MBS, current BMI, type of MBS, time since MBS, regularity of physical activity) and diet-related information (general dietary style, food intolerances) were recorded. Furthermore, two validated scales were included:

1. *Dietary Behavior Inventory-Surgery (DBI-S) *[17]: This scale captures patients’ adherence to the relevant dietary behavior recommendations and guidelines post-MBS. A 5-point response scale to 13 items (e.g., “I always eat in peace without being distracted by anything”), each with two dietary behavior alternatives (unhealthy vs. healthy), is used to rank one’s dietary behavior (*like behavior A/B*). A high DBI-S sum score indicates high adherence (range 13–65). Cronbach’s α was 0.729, indicating acceptable internal consistency [19]. For more details about the DBI-S items, see Table S2.
2. *Patient Health Questionnaire (PHQ-8) *[20]: Eight items assess depression symptomatology. Specifically, they address the frequency of possible depression symptoms (e.g., “feeling down, depressed, or hopeless,” “feeling tired or having little energy”) within the past 2 weeks. On a 4-point Likert scale (with *0* = *not at all* to *3* = *almost every day*), the frequency is rated per item, resulting in a PHQ-8 sum score (range 0–24, cut-off ≥ 10 indicates current depression symptoms). Cronbach’s α was 0.827, indicating good internal consistency [19].

### Statistical Analyses

All statistical analyses were performed at a significance level of *p* = 0.05 and conducted using R [21] and RStudio 4.2.2 [22]. Before performing statistical analyses, data were cleaned according to in-/exclusion criteria, and relevant prerequisites (e.g., linearity, normal distribution of residuals, homoscedasticity, and independence) were checked. The final sample (*N* = 358) was compared with the excluded participants (*n* = 127) for significant differences in their sociodemographic characteristics using *χ*^2^-tests, *t*-tests, and analyses of variance (ANOVA).

First, descriptive analyses for all study variables were calculated. Weight and height were used to calculate participants’ BMI pre- and post-MBS. According to WHO [1, 23], patients’ obesity severity has been classified as BMI 30 ≤ 35 kg/m^2^ (grade I), BMI 35 ≤ 40 kg/m^2^ (grade II), and BMI ≥ 40 kg/m^2^ (grade III). The mean BMI pre- and post-MBS were calculated. Weight outcome after MBS was calculated using the %TWL, for which *%TWL* = *((preoperative body weight − current body weight)/(preoperative body weight))* × *100* was used as a formula [24].

Mean group differences of %TWL and DBI-S for sociodemographic and weight-related variables were calculated using *t*-tests as well as ANOVA, with Tukey’s multiple comparisons serving as post hoc tests. Pearson’s correlation was used to determine significant continuous variables (e.g., age, PHQ-8) as covariates of %TWL and DBI-S.

*F*-test and Tukey’s post hoc tests were calculated to test differences in the DBI-S score since MBS (as monthly grouped blocks). A single linear regression was calculated to test differences in the DBI-S score after MBS (as a continuous monthly variable), using Cohen’s *f* [25] as effect size. *f* = 0.10 corresponds to a weak effect, *f* = 0.25 to a moderate effect, and *f* = 0.40 to a strong effect. *F*-test and Tukey’s post hoc tests were calculated to test differences of the %TWL after MBS (as monthly grouped blocks). A hierarchical regression analysis was performed to examine the interaction between time since MBS and DBI-S on %TWL. *R*^2^ served as the amount of variance explanation of each model. The three regression models consisted of (1) significant covariates and DBI-S, (2) model 1 + time since MBS, and (3) model 2 + interaction of DBI-S and time since MBS. Before this analysis, the DBI-S score was mean-centered to be used as a moderating factor. Cohen’s *f*^2^ [25] was used as effect size, being interpreted: *f*^2^ = 0.02 corresponds to a weak effect, *f*^2^ = 0.15 to a moderate effect, and *f*^2^ = 0.35 to a strong effect. The three models were compared in an ANOVA.

## Results

### Sociodemographic and Weight Characteristics

The mean age of the patients was 44.82 years (SD = 9.85), with a gender distribution of 324 (90.5%) being female and 34 (9.5%) being male. The mean BMI pre-MBS was 50.00 (SD = 7.13, range = 40.04–74.90), while the current mean BMI was 34.72 (SD = 7.41, range = 21.19–64.77). While all patients pre-MBS were classified as obesity grade 3 (*N* = 358, 100.00%), their current medical weight status (i.e., post-MBS) could be classified as normal (*n* = 26, 7.30%), overweight (*n* = 77, 21.50%), obesity grade 1 (*n* = 93, 26.00%), obesity grade 2 (*n* = 74, 20.70%), and obesity grade 3 (*n* = 88, 24.60%). The mean %TWL was 30.11 (SD = 13.41, range = 3.03–60.48). The DBI-S mean sum score was 45.97 (SD = 7.49, range = 22.00–65.00). Regarding PHQ-8, the sample reported a mean sum score of 6.75 (SD = 4.48, range = 0.00–24.00). For more details on the sociodemographic and weight characteristics of the sample, see Table 1.

**Table 1:** Sociodemographic and weight characteristics of the sample as covariates of the adherence to dietary behavior recommendations (DBI-S) and the percentage of total weight loss (%TWL), including significant tests for difference

Covariates	*n*	%	DBI-S		Test for difference_(DBI-S)_		%TWL		Test for difference_(%TWL)_	
			*M*	SD	Test statistic	*p*-value	*M*	SD	Test statistic	*p-*value
Age (in years)	358	100.00	45.97	7.49	*r* = .13	.017*	30.11	13.41		
Gender										
Female	324	90.50	46.10	7.50			29.95	13.54		
Male	34	9.50	44.68	7.31			31.62	12.12		
Marital status										
Married	215	60.10	46.30	7.51			30.76	12.98		
Partnership	58	16.20	44.24	8.34			31.63	14.01		
Single	85	23.70	46.31	6.55			27.44	13.89		
Educational degree										
Without degree	5	1.40	40.40	8.93			30.08	9.42		
School degree	283	79.00	45.69	7.57			30.04	13.81		
University degree	57	15.90	47.25	6.66			31.19	12.05		
Other	13	3.60	48.61	5.91			27.11	12.93		
Occupational status										
Not employed	28	7.80	46.46	7.07			29.33	12.67		
Employed	273	76.30	45.77	7.55			30.16	13.39		
Retired	30	8.40	46.30	7.45			31.87	16.52		
Other	27	7.50	47.11	7.73			29.78	10.75		
Physical activity					*F*_(4,353)_ = 10.05	< .001***				
Always	88	24.58	49.3	6.65			30.41	13.40		
Mostly	102	28.49	46.8	6.56			30.27	13.08		
Sometimes	73	20.39	44.3	7.41			30.87	13.18		
Rarely	62	17.32	43.6	7.92			30.14	12.95		
Never	33	9.27	42.4	7.88			27.13	15.98		
PHQ-8	358	100.00	45.97	7.49	*r* = − .25	< .001***	30.11	13.41		
BMI pre-MBS	358	100.00	45.97	7.49			30.11	13.41	*r* = .23	< .001***
Type of MBS									*F*_(3,354)_ = 4.42	.005**
Sleeve gastrectomy	196	54.75	46.62	7.55			27.78	13.27		
Roux-en-Y gastric bypass	125	34.92	44.91	7.61			33.10	13.15		
Omega loop bypass	30	8.38	45.97	6.90			32.46	13.21		
Other technique^a^	7	1.96	46.71	4.50			32.01	12.49		

*N* = 358

*N* sample, *n* subsample, *%* percentual frequency, *M* mean, *SD* standard derivation, *BMI* body mass index, *DBI-S* adherence to dietary behavior recommendations, *%TWL* percentage of total weight loss, *MBS* metabolic and bariatric surgery, *PHQ-8* depression symptomatology

^*^p < 0.05, **p < 0.01, ***p < 0.001

^a^4 of the 7 subjects provided more information in the optional free text field, namely, “sleeve beforehand, then conversion bypass,” “mini gastric bypass,” “banded sleeve,” and “endoscopic gastric reduction”

### Determinants of Adherence to Dietary Behavior Recommendations (DBI-S) and %TWL (Covariates)

Significance tests for the difference of DBI-S excluded gender, marital status, educational degree, occupational status, and BMI pre-MBS as determinants of DBI-S (*p* > 0.05). Age, regularity of physical activity, and PHQ-8 were identified as significant determinants of adherence, see Table 1 and Fig. S1.

Significance tests for the difference of %TWL excluded age, gender, marital status, educational degree, occupational status, regularity of physical activity, and the PHQ-8 score as determinants of %TWL (*p* > 0.05). Type of MBS and BMI pre-MBS were identified as significant covariates of %TWL, see Table 1 and Fig. S1.

### Adherence to Dietary Behavior Recommendations (DBI-S) After MBS

**Fig. 1:**
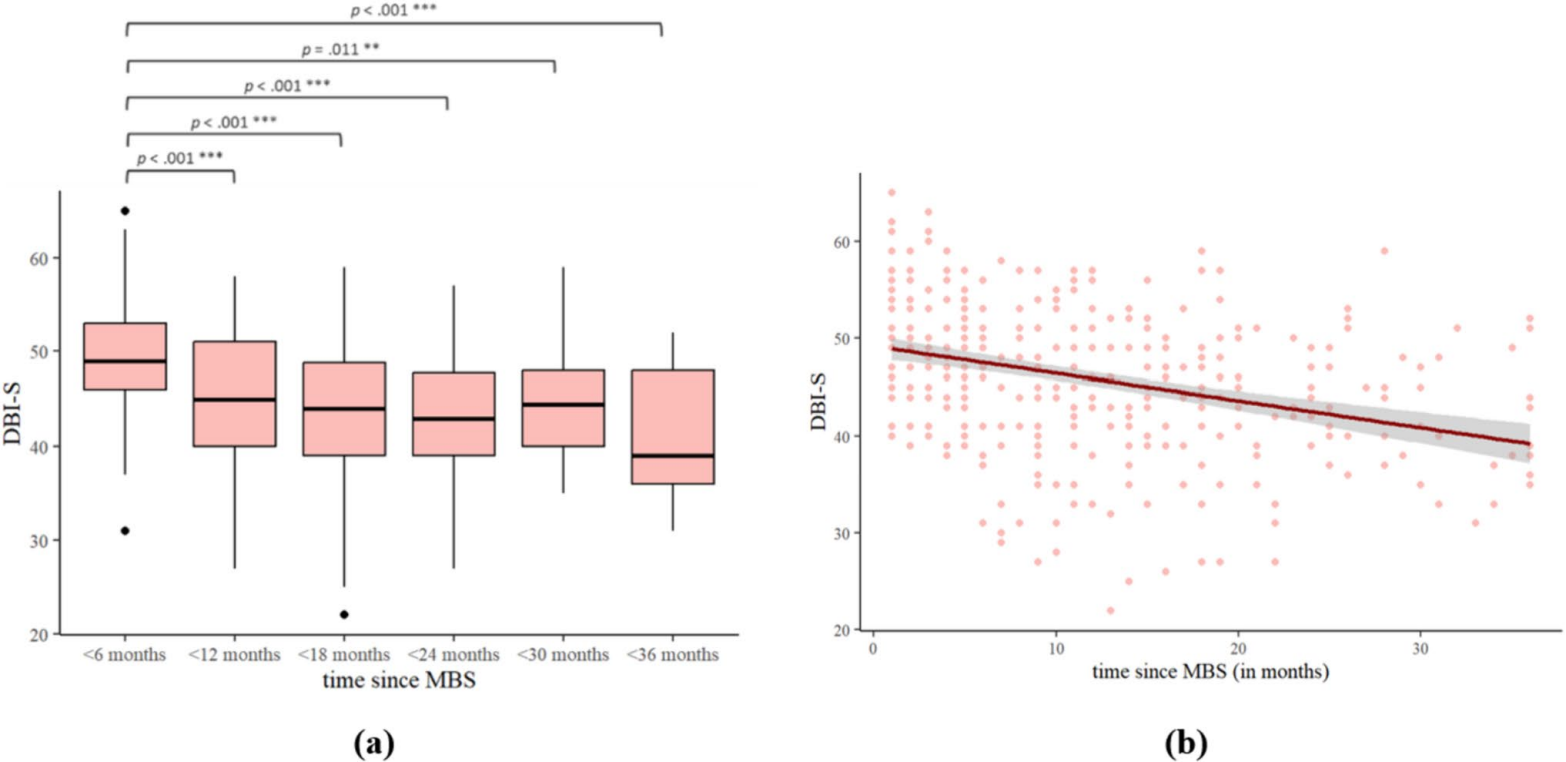
*N* = 358. *p < 0.05, **p < 0.01, ***p < 0.001. *N* = sample; *p* = *p*-value; DBI-S = adherence to dietary behavior recommendations; MBS = metabolic and bariatric surgery. **a** *F*-test and Tukey’s post hoc tests were used as significant tests of difference. Shown above is the patients’ DBI-S since MBS, *F*_(5,352)_ = 12.35, *p* < .001. Time since MBS is calculated in monthly blocks: < 6 months (*n* = 140), < 12 months (*n* = 81), < 18 months (*n* = 58), < 24 months (*n* = 34), < 30 months (*n* = 28), < 36 months (*n* = 17). **b** Shown above is the single linear regression line, including the standard derivation, of the DBI-S since MBS (in months), *R*.^2^ = .12, *F*_(1,356)_ = 47.85, *p* < .001

The difference in DBI-S score for time since MBS calculated in monthly blocks was significant, *F*_(5,352)_ = 12.35, *p* < 0.001, see Fig. 1a. If the time since MBS (calculated continuously) increases by one unit (1 month), the DBI-S score decreases by − 0.28 units, *b* =  − 0.28, *t*_(358)_ =  − 6.92, *p* < 0.001. Twelve percent of the variance of the DBI-S score is explained by the time since MBS, *F*_(1,356)_ = 47.85, *p* < 0.001 (Cohen’s *f* = 0.37), indicating a moderate effect size, see Fig. 1b.

### Associations Between Time Since MBS and %TWL, Moderated by Adherence to Dietary Behavior Recommendations (DBI-S)

**Fig. 2:**
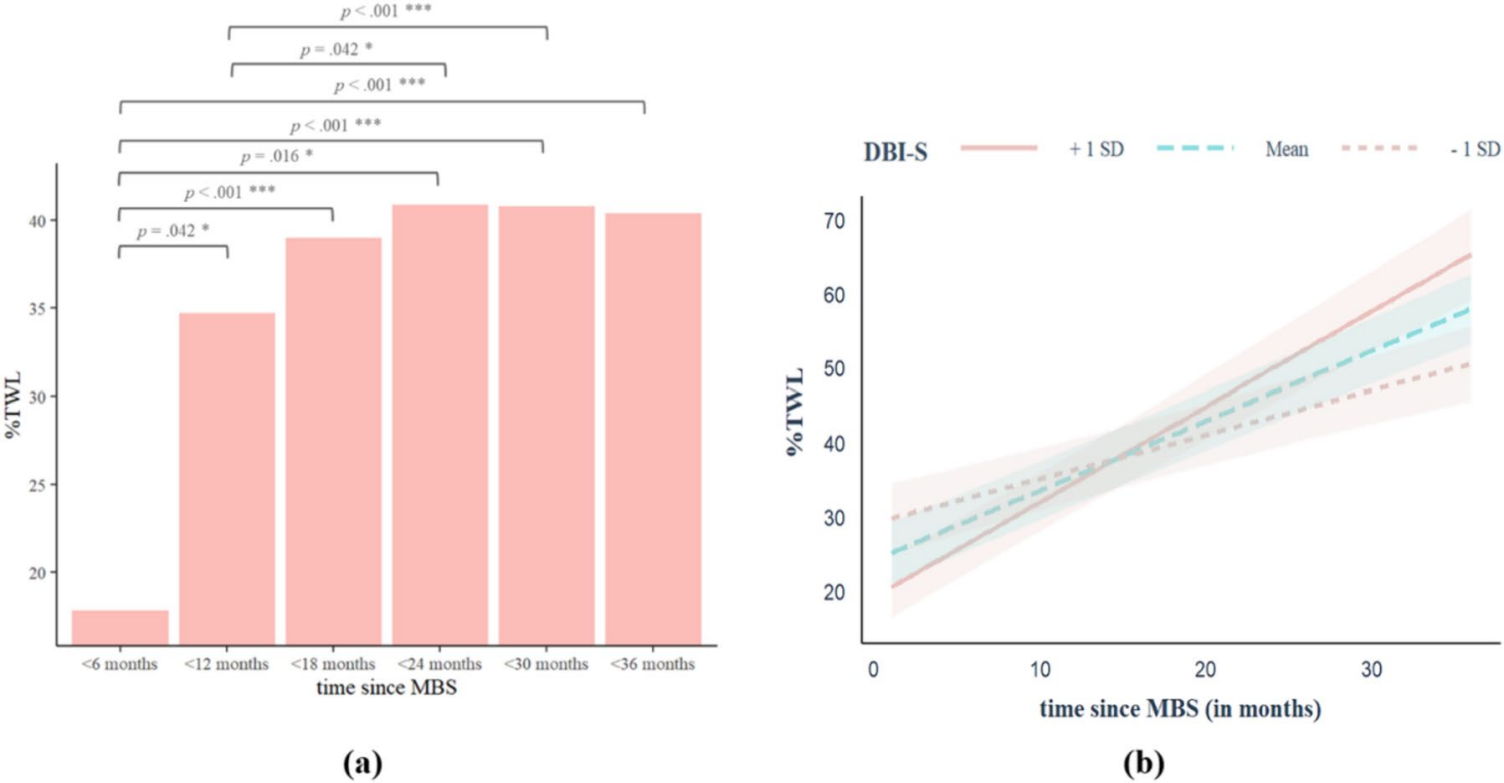
*N* = 358. *p < 0.05, **p < 0.01, ***p < 0.001. *N* = sample; *p* = *p*-value; SD = standard derivation; DBI-S = adherence to dietary behavior recommendations; %TWL = percentage of total weight loss; MBS = metabolic and bariatric surgery. **a** *F*-test and Tukey’s post hoc tests were used as significant tests of difference. Shown above is the patients’ %TWL grouped by time since MBS, *F*_(5,352)_ = 92.75, *p* < .001. Time since MBS is calculated in monthly blocks: < 6 months (*n* = 140), < 12 months (*n* = 81), < 18 months (*n* = 58), < 24 months (*n* = 34), < 30 months (*n* = 28), < 36 months (*n* = 17). **b** Shown above is the interaction effect of time since MBS and %TWL, moderated by DBI-S. The DBI-S mean sum score was 45.97 (SD = 7.49)

The greatest difference in %TWL was reported by patients within the first 12 months after MBS, see Fig. 2a. There was a significant interaction effect between time since MBS and DBI-S score, see Table 2. Results show that the DBI-S score moderated the effect between time since MBS and %TWL significantly. The effect size of model 3 (see Table 2) indicates a strong effect. All significant determinants (covariates) of adherence and %TWL were considered. The visualization of the interaction effect shows that a higher DBI-S score was associated with a higher %TWL after MBS, see Fig. 2b.

**Table 2:** Summary of hierarchical regression analysis of variables predicting the percentage total weight loss (%TWL)

Predictors	Model 1				Model 2				Model 3			
	*B*	SD	*t*	*p*	*B*	SD	*t*	*p*	*B*	SD	*t*	*p*
DBI-S score	− 0.58	0.10	− 6.11	< .001***	− 0.16	0.08	− 1.91	.056	− 0.67	0.12	− 5.75	< .001***
Time since MBS					0.89	0.06	14.34	< .001***	− 1.17	0.35	− 3.32	< .001***
DBI-S: time since MBS									0.05	0.01	5.93	< .001***
Adjusted *R*^2^	17.21%				47.97%				52.65%			
Test statistic	*F*_(11,346)_ = 7.75, *p* < .001***				*F*_(12,345)_ = 28.43, *p* < .001***				*F*_(13,344)_ = 31.54, *p* < .001***			
Cohen’s *f*^2^	0.21				0.92				1.11			
Test statistic for change in *R*^2^					*F*_(1,345)_ = 225.91, *p* < .001***				*F*_(1,344)_ = 35.12, *p* < .001***			

*N* = 358. Each model included the relevant covariates “type of MBS,” “age,” “regularity of physical activity,” and “PHQ-8,” see Table S3 for a detailed summary including all covariates

*N* sample, *B* coefficient *B*, *SD* standard derivation, *t t*-value, *p p*-value, *R*^*2*^ variance explained, *Cohen’s f*^*2*^ effect size, *DBI-S* adherence to dietary behavior recommendations, *MBS* metabolic and bariatric surgery, *%TWL* percentage total weight loss, *PHQ-8* depression symptomatology

^*^p < 0.05, **p < 0.01, ***p < 0.001

## Discussion

The prevalence of people suffering from obesity is a globally growing health problem [1]. MBS represents the gold standard in treating severe obesity [4, 5]. However, MBS’ success has some shortcomings (e.g., weight regain) [7, 9].

The present study aimed to understand the relationship between adherence to dietary behavior recommendations and %TWL after MBS. Within 6 months after MBS, adherence seemed to peak, stabilizing at a lower level afterward. Adherence and time since MBS were associated with %TWL. In detail, a high adherence was related to a higher %TWL after MBS. Overall, adherence to dietary behavior recommendations seems crucial for maximizing MBS’ success permanently.

Firstly, this study investigated how adherence to dietary behavior recommendations has changed over time since MBS. Within 6 months after MBS, the adherence seemed to be at its peak. In the following months, it dropped, stabilizing at a lower level. Consequently, patients seem to adhere to their new dietary plan for a specific period but cannot permanently implement dietary behavior recommendations into their daily lives. According to previous research [8, 26–28], the first 12 months seem crucial for determining the MBS’ success over time. Willett [29] concluded that a healthy lifestyle, which is defined as a combination of a healthy diet, engaging in moderate activity, avoiding excess weight, and not smoking, is the most beneficial for one’s health—only for percent attained this healthy lifestyle [29]. Burgess et al. [30] also summarized that lifestyle change programs fail due to low adherence. Many patients might struggle to adhere to dietary behavior recommendations because there is no direct success. Sarwer et al. [13] described the potential reasons for negative outcomes in the first years after MBS. Although 20–30% of all patients are affected, the reasons for these negative outcomes have not been fully identified yet. If adherence is essential for MBS’ success, maximizing adherence and preventing its drop 6 months post-MBS should be aimed. This implies adapting the aftercare management more closely to the patients’ individual needs. MBS requires lifelong aftercare [4, 5], in which patients could receive more support and involvement (e.g., frequent follow-up appointments over a longer period, electronic lifestyle-tracking applications).

Secondly, this study investigated the role of adherence to dietary behavior recommendations on patients’ %TWL after MBS. High adherence was related to a higher %TWL. Several findings also implicate that maintaining one’s weight after a successful weight loss depends on high adherence to behavior recommendations post-MBS [12, 13, 31]. In a study with the majority of patients (> 80%) reporting weight regain after MBS (laparoscopic sleeve gastrectomy), the reasons for this development were reported [32]. Weight regain was justified by approximately half of these patients with non-adherence to the recommended guidelines [32]. In particular, lack of exercise, not meeting with a dietitian, and inefficiently controlling dietary behavior were reported as reasons [32]. Revisional MBS patients tended to have greater deficits and were less satisfied with the procedure [32]. According to their %TWL (< 25%), these patients might not be categorized as “good responders” to MBS [33]. The identified covariates in the present study are consistent with Herman et al. [31], who examined physical activity post-MBS, controlling for age, type of MBS, and time since MBS. Depression symptomatology is also relevant for adherence since obesity combined with appearance concerns, and depressive moods are associated with lower adherence (regarding physical activity) [34]. BMI pre-MBS was also a determinant of %TWL. Although only patients with obesity grade 3 took part in the present study, this finding was evident. Considering and comparing patients of all three obesity grades in the future might accelerate this finding.

In a systematic review, both the drivers and barriers to adherence were summarized [30]. The drivers identified were succeeding earlier in weight loss, having a lower BMI pre-MBS and better mood, being male and of older age, while lacking motivation, experiencing environmental, societal and social burdens, lack of time, having health limitations (mental or physical), having negative thoughts/moods, being socioeconomically restricted, knowledge/awareness gaps, and lacking joy in physical activity represented barriers to a successful change in lifestyle [30]. Special attention should also be paid to having negative moods (e.g., depression symptoms) and unrealistic %TWL expectations [30].

In the future, taking into account adherence, its drivers and barriers might emphasize optimization of the outcomes [30]. The findings of this study support and extend this conclusion. Considering and integrating the relationship between adherence to dietary behavior recommendations and %TWL after MBS into the aftercare of patients might sustainably optimize MBS’ outcomes.

### Limitations

Some limitations must be considered when interpreting the study results. Firstly, it was a cross-sectional study design. The results cannot be understood causally. Despite querying the adherence to dietary behavior recommendations and the BMI over time, this took place at a single point in time. Accordingly, recall biases cannot be ruled out. In addition, there was heterogeneity in the sample regarding the time since MBS. The statistical analyses established a causal model based on theoretical principles/previous findings. Nevertheless, it should be noted that neither correlation nor regression proves causality between constructs [35]. Instead, the present study provides preliminary work (including theoretically derived causality) that must be tested in a longitudinal design for causal conclusions in the future.

Furthermore, it was an online survey that had to be completed in self-report. Therefore, selection as well as self-report biases might occur. Although a web-based study is economical in terms of time and costs, it is uncertain whether and, if so, which patients did not take part in the study. In the future, a different survey medium could be chosen to increase the results’ generalizability. However, the number of Internet and social media users implies that the vast majority of the population has Internet access and uses social media [36]. The self-report was counteracted with anonymization. Despite the limitations associated with self-report, it offers the opportunity to survey patients despite inconsistent/differing aftercare and potentially varying aftercare attendance. Although self-report is a purely subjective measure, it also provides insights into aspects that cannot be objectively measured. In the future, it could be supplemented by objective measures (e.g., behavioral observation, aftercare appointments).

Furthermore, the gender distribution of the sample (90.5% female, 9.5% male) has to be considered. Due to the very high proportion of women, there is a bias compared to the “normal BMI > 40 distribution” [37]. Future studies should try to assess an even more representative sample. Additionally, ethnicity was not recorded. Regarding the sociodemographic variables of the final sample and the excluded participants, there was a significant difference in their occupational status. However, it is not believed that this had an impact on the present results.

Since this study did not distinguish between primary and revisional MBS, future research should address this. However, the comparison of long-term (> 5–15 years) outcomes between primary and revisional MBS (laparoscopic sleeve gastrectomy) showed no significant differences in weight loss or related medical outcomes [32]. It also seems particularly interesting whether the type of aftercare management correlates with or even influences adherence. However, which aftercare the participants received and used was not assessed. Since aftercare for MBS is not uniformly regulated, assessing it represents a challenge. Only patients until 3 years from MBS were included to avoid heterogeneous outcomes. In future studies, the influence of adherence to dietary behavior recommendations on %TWL even 3 years after MBS should be examined.

Since the DBI-S [17] is the first and comparatively new measure for assessing adherence to dietary behavior recommendations, there are no studies for further contextualization of the results in research. However, the present study is one of the first to lay the foundation for this.

## Conclusions

Overall, this study confirms a relationship between time since MBS, adherence to dietary behavior recommendations, and %TWL after MBS. Hence, implications arise for optimizing MBS’ success and for its application in aftercare management. It was shown that adherence plays a crucial role in MBS’ initial success, peaking 6 months post-surgically and decreasing afterward. Solutions for preventing this drop should be identified in the future. For the success of the MBS, it also intuitively turned out that higher adherence to dietary behavior recommendations is related to a higher %TWL. A preoperative patient education that tapers off in MBS’ aftercare management and leaves patients more to their personal responsibility does not seem to be the solution. Therefore, patient-oriented options must be created to increase adherence post-operatively since adherence is relevant for optimizing MBS’ success.

### Supplementary Information

Below is the link to the electronic supplementary material.

Supplementary file1 (PDF 396 KB)

## Data Availability

The data supporting the results presented in this article is available upon reasonable request to the corresponding author.

## Abbreviations

BMI: Body mass index
DBI-S: Dietary Behavior Inventory-Surgery (measuring patients’ adherence to the dietary behavior recommendations and guidelines post-MBS)
MBS: Metabolic and bariatric surgery
PHQ-8: Patient Health Questionnaire (assessing patients’ depression symptomatology)
%TWL: Percentage total weight loss
WHO: World Health Organization
