# Supplementary material for: Adherence to Dietary Behavior Recommendations Moderates the Effect Between Time Since Metabolic and Bariatric Surgery and Percentage Total Weight Loss
Source: Obes Surg. 2024 Jun 18;34(8):2930–9. doi: 10.1007/s11695-024-07359-2 (PMC11289064; doi:10.1007/s11695-024-07359-2)
Supplement: Supplementary file 1 — Supplementary file1 (PDF 396 KB) [file 11695_2024_7359_MOESM1_ESM.pdf]

## Supplementary Materials

***Figures***

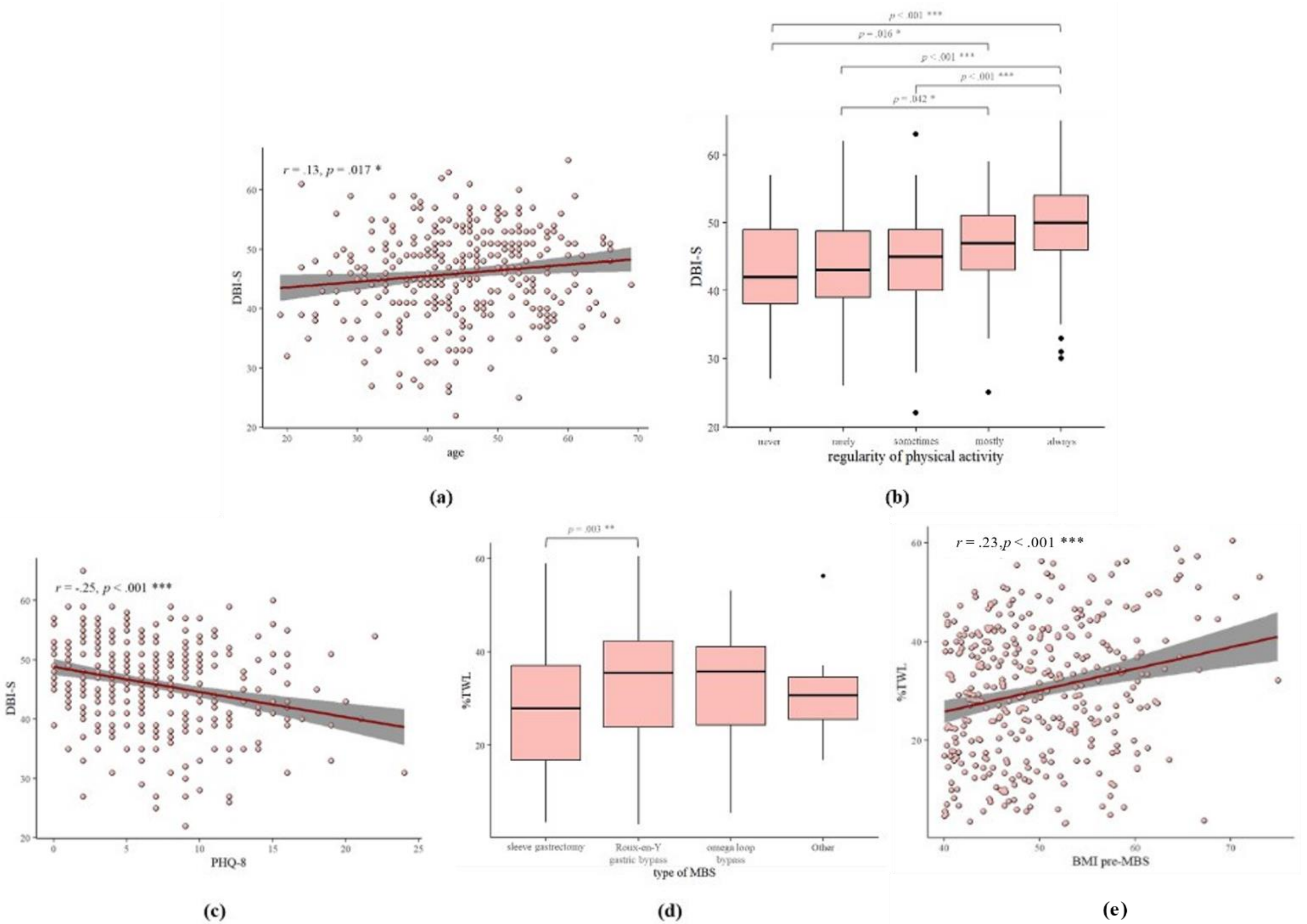


**Figure S1.** Covariates of adherence to dietary behavior recommendations (DBI-S) and percentage total weight loss (%TWL). *F*-test and Tukey post-hoc tests were used as significant tests of difference. $N = 358$. * $p < 0.05$, ** $p < 0.01$, *** $p < 0.001$. $N$ = sample; DBI-S = Adherence to Dietary Behavior Recommendations; %TWL = Percentage of Total Weight Loss; MBS = Metabolic and Bariatric Surgery; PHQ-8 = depression symptomatology. **(a)** Age was significantly correlated with DBI-S, $r = .13$, $p = .017$. **(b)** Shown above is the patients' DBI-S grouped by their regularity of physical activity, $F_{(4,353)} = 10.05$, $p < .001$. Categories of regularity of physical activity: never ($n = 33$), rarely ($n = 62$), sometimes ($n = 73$), mostly ($n = 102$), always ($n = 88$). **(c)** PHQ-8 was significantly correlated with DBI-S, $r = -.25$, $p < .001$. **(d)** Shown above is the patients' %TWL grouped by their type of MBS, $F_{(3,354)} = 4.54$, $p = .004$. Type of MBS: sleeve gastrectomy ($n = 212$), Roux-en-Y gastric bypass ($n = 131$), omega loop bypass ($n = 30$), other ($n = 7$). **(e)** BMI pre-MBS was significantly correlated with %TWL, $r = .23$, $p < .001$.

## *Tables*

**Table S1**

Sociodemographic characteristics of the final sample and all excluded participants, including significant tests for difference

| | final sample ($N$ = 358) | | | | excluded participants ($n$ = 127) [a] | | | | Test for difference (final sample vs. excluded participants) | |
|---|---|---|---|---|---|---|---|---|---|---|
| Covariates | *n* | % | *M* | *SD* | *n* | % | *M* | *SD* | *Test statistic* | *p* value |
| Age (in years) | 358 | 100.00 | 44.82 | 9.85 | 120 | 94.49 | 44.54 | 10.49 | $\chi^2$ (48) = 36.40 | .890 |
| Gender [b] | | | | | | | | | $F_{(1, 476)}$ = 0.47 | .495 |
| Female | 324 | 90.50 | | | 106 | 83.46 | | | | |
| Male | 34 | 9.50 | | | 14 | 11.02 | | | | |
| Marital status [c] | | | | | | | | | $F_{(5,472)}$ = 1.11 | .354 |
| Married | 215 | 60.10 | | | 73 | 57.48 | | | | |
| Partnership | 58 | 16.20 | | | 17 | 13.39 | | | | |
| Single | 85 | 23.70 | | | 29 | 22.83 | | | | |
| Educational degree [c] | | | | | | | | | $F_{(7,470)}$ = 1.10 | .359 |
| Without degree | 5 | 1.40 | | | 0 | 0.00 | | | | |
| School degree | 283 | 79.00 | | | 103 | 81.10 | | | | |
| University degree | 57 | 15.90 | | | 12 | 9.45 | | | | |
| Other | 13 | 3.60 | | | 5 | 3.94 | | | | |
| Occupational status [c] | | | | | | | | | $F_{(6,471)}$ = 2.20 | .042 * |
| Not employed | 28 | 7.80 | | | 10 | 7.84 | | | | |
| Employed | 273 | 76.30 | | | 81 | 63.78 | | | | |
| Retired | 30 | 8.40 | | | 17 | 13.39 | | | | |
| Other | 27 | 7.50 | | | 12 | 9.45 | | | | |

*Note*. $N$ = 358. * $p < 0.05$, ** $p < 0.01$, *** $p < 0.001$. $N$ = sample; $n$ = subsample; % = percentual frequency; $M$ = mean; $SD$ = standard derivation. [a] The data set of excluded participants also includes "not available", so that 127 does not always represent the sum of all answers. [b] Although gender only has two categories, the data set of excluded participants also includes the categories “diverse” and “not available”. [c] For simplicity, some answer categories have been combined.

**Table S2**

Items and Scoring of the Dietary Behaviour Inventory-Surgery (DBI-S)

| Items | Behavior A | Scoring | | | | | Behavior B |
|---|---|---|---|---|---|---|---|
| 1 | Ich esse immer in Ruhe, ohne dass ich von irgendetwas abgelenkt werde.<br>*I always eat in peace, without being distracted by anything.* | 5 | 4 | 3 | 2 | 1 | Während des Essens beschäftige ich mich immer mit anderen Themen (z.B. Fernsehen, Gedanken an die Arbeit, Lesen).<br>*While eating, I always occupy myself with other topics (e.g. watching TV, thinking about work, reading).* |
| 2 | Bei der Einnahme meiner Mahlzeiten lasse ich mir immer Zeit.<br>*I always take my time when eating my meals.* | 5 | 4 | 3 | 2 | 1 | Meine Mahlzeiten nehme ich immer schnell ein.<br>*I always eat my meals quickly.* |
| 3 | Beim Essen nehme ich kleine Bissen, die ich lange durchkaue. | 5 | 4 | 3 | 2 | 1 | Ich nehme beim Essen große Bissen, die ich nur kurz kaue. |

| | | | | | | | |
|---|---|---|---|---|---|---|---|
| | *When I eat, I take small bites and chew them for a long time.* | | | | | | *When I eat, I take large bites and only chew them briefly.* |
| 4 | Ich halte immer Getränkepause vor den Mahlzeiten (15 Minuten) und nach den Mahlzeiten (30 Minuten) ein.<br>*I always take a drink break before meals (15 minutes) and after meals (30 minutes).* | 5 | 4 | 3 | 2 | 1 | Ich nehme während der Mahlzeiten Getränke zu mir.<br>*I drink drinks during meals.* |
| 5 | Ich nehme täglich mindestens 1,5 Liter Flüssigkeit (ausgenommen alkoholische Getränke bzw. zucker-/süßungsmittelhaltige/kohlensäurehaltige Getränke) zu mir.<br>*I consume at least 1.5 liters of liquid (excluding alcoholic drinks or drinks containing sugar/sweeteners/carbonated drinks) every day.* | 5 | 4 | 3 | 2 | 1 | Ich nehme täglich deutlich weniger als 1,5 Liter Flüssigkeit (ausgenommen alkoholische Getränke bzw. zucker-/süßungsmittelhaltige/kohlensäurehaltige Getränke) zu mir.<br>*I consume significantly less than 1.5 liters of liquid every day (excluding alcoholic drinks or drinks containing sugar/sweeteners/carbonated drinks).* |
| 6 | Ich nehme täglich und in empfohlener Menge ein hochwertiges Eiweißpräparat zu mir.<br>*I take a high-quality protein supplement daily and in the recommended amount.* | 5 | 4 | 3 | 2 | 1 | Ich nehme nie Eiweißpräparate zu mir.<br>*I never take protein supplements.* |
| 7 | Ich esse täglich Gemüse.<br>*I eat vegetables every day.* | 5 | 4 | 3 | 2 | 1 | Ich esse nie Gemüse.<br>*I never eat vegetables.* |
| 8 | Ich esse täglich Obst.<br>*I eat vegetables every day.* | 5 | 4 | 3 | 2 | 1 | Ich esse nie Obst.<br>*I never eat fruit.* |
| 9 | Ich esse nie Süßigkeiten (z.B. Schokolade, Kekse, Gebäck).<br>*I never eat sweets (e.g. chocolate, cookies, pastries).* | 5 | 4 | 3 | 2 | 1 | Ich esse täglich Süßigkeiten (z.B. Schokolade, Kekse, Gebäck).<br>*I eat sweets every day (e.g. chocolate, cookies, pastries).* |
| 10 | Ich esse grundsätzlich Vollkornprodukte (z.B. Vollkornnudeln, Vollkornbrot).<br>*I generally eat whole grain products (e.g. whole grain pasta, whole grain bread).* | 5 | 4 | 3 | 2 | 1 | Ich esse grundsätzlich Weißmehlprodukte (z.B. „herkömmliche" Nudeln, Weißbrot/Graubrot).<br>*I generally eat white flour products (e.g. "conventional" pasta, white bread/gray bread).* |
| 11 | Ich trinke nie zucker-/ süßungsmittelhaltige Getränke (z.B. Säfte, Limonade, gesüßter Kaffee/Tee, Zero-Getränke).<br>*I never drink drinks that contain sugar/sweeteners (e.g. juices, lemonade, sweetened coffee/tea, zero drinks).* | 5 | 4 | 3 | 2 | 1 | Ich trinke täglich zucker-/ süßungsmittelhaltige Getränke (z.B. Säfte, Limonade, gesüßter Kaffee/Tee, Zero-Getränke).<br>*I drink drinks containing sugar/sweeteners every day (e.g. juices, lemonade, sweetened coffee/tea, zero drinks).* |
| 12 | Ich esse täglich Lebensmittel mit vielen Kalorien, wie z.B. Smoothies, Eiscreme, Milchshakes, Säfte, Schokolade, Kuchen, Kekse.<br>*I eat high-calorie foods every day, such as smoothies, ice cream, milkshakes, juices, chocolate, cakes, cookies.* | 1 | 2 | 3 | 4 | 5 | Ich esse nie Lebensmittel mit vielen Kalorien, wie z.B. Smoothies, Eiscreme, Milchshakes, Säfte, Schokolade, Kuchen, Kekse.<br>*I never eat foods with a lot of calories, such as smoothies, ice cream, milkshakes, juices, chocolate, cakes, cookies.* |
| 13 | Ich esse nie Fast-Food- und Fertigprodukte (z.B. Tiefkühlpizza, Mikrowellenfertiggerichte).<br>*I never eat fast food and ready-made products (e.g. frozen pizza, microwave ready meals).* | 5 | 4 | 3 | 2 | 1 | Ich esse täglich Fast-Food- und Fertigprodukte (z.B. Tiefkühlpizza, Mikrowellenfertiggerichte).<br>*I eat fast food and convenience foods (e.g. frozen pizza, microwave ready meals) every day.* |

*Note.* The items are presented in both German (original) and English versions (for publication only).

**Table S3**

Summary of hierarchical regression analysis of variables predicting the percentual total weight loss (%TWL)

| | Model 1 | | | | Model 2 | | | | Model 3 | | | |
|---|---|---|---|---|---|---|---|---|---|---|---|---|
| Predictors | *B* | *SD* | *t* | *p* | *B* | *SD* | *t* | *p* | *B* | *SD* | *t* | *p* |
| Type of MBS[a] | | | | | | | | | | | | |
| Sleeve gastrectomy | -4.68 | 2.44 | -1.92 | .056 | -2.31 | 1.95 | -1.19 | .236 | -3.06 | 1.86 | -1.65 | .100 |
| Roux-en-Y gastric bypass | 0.38 | 2.52 | 0.15 | .881 | 0.82 | 2.00 | 0.41 | .683 | -0.27 | 1.91 | -0.14 | .886 |
| Other technique | -2.01 | 5.17 | -0.39 | .698 | -2.30 | 4.10 | -0.56 | .575 | -3.84 | 3.92 | -0.98 | .328 |
| BMI pre-MBS[a] | 0.48 | 0.09 | 5.16 | <.001*** | 0.22 | 0.08 | 2.89 | .004** | 0.20 | 0.07 | 2.82 | .005** |
| Age (in years)[b] | 0.13 | 0.07 | 1.86 | .064 | -0.05 | 0.06 | -0.88 | .377 | -0.06 | 0.05 | -1.06 | .290 |
| Regularity of physical activity[b] | | | | | | | | | | | | |
| Mostly | -3.01 | 1.81 | -1.67 | .097 | -1.84 | 1.43 | -1.28 | .200 | -1.67 | 1.37 | -1.22 | .224 |
| Sometimes | -3.21 | 1.99 | -1.61 | .108 | -3.19 | 1.58 | -2.02 | .045* | -2.41 | 1.51 | -1.59 | .112 |
| Rarely | -4.69 | 2.11 | -2.22 | .027* | -4.38 | 1.68 | -2.59 | .010* | -3.55 | 1.60 | -2.22 | .027* |
| Never | -8.56 | 2.61 | -3.28 | .001** | -5.90 | 2.08 | -2.84 | .005** | -5.00 | 1.99 | -2.52 | .012* |
| PHQ-8[b] | -0.40 | 0.15 | -2.65 | .008** | -0.21 | 0.12 | -1.72 | .086 | -0.20 | 0.11 | -1.72 | .086 |
| DBI-S score | -0.58 | 0.10 | -6.11 | <.001*** | -0.16 | 0.08 | -1.91 | .056 | -0.67 | 0.12 | -5.75 | <.001*** |
| Time since MBS | | | | | 0.89 | 0.06 | 14.34 | <.001*** | -1.17 | 0.35 | -3.32 | <.001*** |
| DBI-S: Time since MBS | | | | | | | | | 0.05 | 0.01 | 5.93 | <.001*** |
| Adjusted $R^2$ | 17.21% | | | | 47.97% | | | | 52.65% | | | |
| Test statistic | $F_{(11,346)} = 7.75$, $p < .001$*** | | | | $F_{(12,345)} = 28.43$, $p < .001$*** | | | | $F_{(13,344)} = 31.54$, $p < .001$*** | | | |
| Cohen's $f^2$ | 0.21 | | | | 0.92 | | | | 1.11 | | | |
| Test statistic for change in $R^2$ | | | | | $F_{(1,345)} = 225.91$, $p < .001$*** | | | | $F_{(1,344)} = 35.12$, $p < .001$*** | | | |

*Note.* $N = 358$. * $p < 0.05$, ** $p < 0.01$, *** $p < 0.001$. *N* = sample; *B* = Coefficient *B*; *SD* = standard derivation; *t* = *t*-value; *p* = *p* value; $R^2$ = variance explained; Cohen's $f^2$ = effect size; BMI = Body Mass Index; DBI-S = Adherence to Dietary Behavior Recommendations; MBS = Metabolic and Bariatric Surgery; %TWL = Percentual Total Weight Loss; PHQ-8 = depressive symptoms; [a] = covariates of %TWL; [b] = covariates of DBI-S.
